# Plasmonic metafiber for all-fiber *Q*-switched cylindrical vector lasers

**DOI:** 10.1515/nanoph-2022-0696

**Published:** 2023-02-14

**Authors:** He Hua, Chao Zeng, Zhiwen He, Hua Lu, Yueqing Du, Dong Mao, Jianlin Zhao

**Affiliations:** Key Laboratory of Light Field Manipulation and Information Acquisition, Ministry of Industry and Information Technology, Shaanxi Key Laboratory of Optical Information Technology, School of Physical Science and Technology, Northwestern Polytechnical University, Xi’An 710129, China

**Keywords:** cylindrical vector beam, fiber laser, metafiber, metasurface, structured laser

## Abstract

Metafibers, by integrating metasurface at the optical fiber tip, are emerging as the significant optical coupling platforms for nanophotonics and fiber-optic communities. Here, we propose a plasmonic metafiber for converting the fundamental mode to first-order mode in fiber, and as proof of device performance, demonstrate an all-fiber *Q*-switched cylindrical vector laser using the metafiber. Based on polarization-dependent plasmonic resonance, a polarization-independent mode conversion metasurface is designed theoretically and numerically, fabricated directly on fiber facet, and packaged as an all-fiber component with efficiency up to 21% at 1550-nm band. Using the metafiber in an all-fiber laser, *Q*-switched azimuthally polarized beam (APB) and radially polarized beam (RPB) are delivered at wavelength of 1548.5 nm with pulse durations from ∼7 to ∼2 μs when pump power increases from 30 to 120 mW. The mode purities of the APB and RPB are 86.5% and 90.7%, respectively. This work outlines a new strategy to integrate metasurfaces into “all-in-fiber” systems and offers a reliable route to construct next-generation laser sources, such as all-fiber ultrafast structured lasers.

## 1 Introduction

Metasurfaces, by adjusting the shape, size, and spatial arrangement parameters of the meta-atom, can manipulate the phase, amplitude, and polarization of the light field at the subwavelength scale [1]. In the past decade, a great number of novel devices and functionalities based on metasurfaces were demonstrated to modulate light field in spatial and/or temporal domains, including metalens [2, 3], holographic display [4, 5], structured light generation [6, 7], absorber [8], pulse shaping [9], and nonlinear optics [10]. Although metasurfaces show unprecedented capabilities to manipulate light field, their ultrasmall footprint is a double-edged sword: on the one hand, miniaturizing the optical devices on chips; on the other hand, not compatible with traditional bulk optical elements and systems. Besides exploiting new functionalities and empowering dynamic tunability, currently, integrating metasurfaces with traditional optoelectronic devices and systems is emerging as a significant motive force to promote the engineering and industrial applications of metasurfaces [11–16]. With the assistance of increasingly advanced micro-/nano-manufacturing technologies, metasurfaces have been fabricated directly on fiber tips [17], semiconductor lasers [18], and CMOS cameras [19]. Amongst, due to the excellent potentials for infusing new life to “lab-on-fiber,” metasurfaces on fiber tips, usually called metafibers, have attracted intense attentions in recent years [20–24]. In 2022, Zhang et al. reported the first demonstration of metafiber-based saturable absorbers (SAs) for ultrafast all-fiber lasers at operation wavelengths of 1.5 and 2 μm [25]; Gu et al. proposed a large-scale fabrication process, *i.e.* modified self-assembly nanosphere lithography, to directly fabricate metasurface-SA onto optical fiber tip, and demonstrated an all-fiber picosecond soliton mode-locked laser [26]. These pioneering works prove the feasibilities, advantages, and potentials of metafibers for temporal-domain light field manipulations in all-fiber lasers.

In the spatial domain, a series of all-fiber mode converters based on mode coupling theory have been proposed to generate the cylindrical vector beams (CVBs) and vortex beams in fiber lasers [27], such as offset-spliced fibers (OSFs) [28], long period fiber gratings (LPFGs) [29], and mode-selective couplers (MSCs) [30]. In 2015, Xu et al. demonstrated a radially polarized mode-locked fiber laser with pulse duration from 2.8 to 23 ns using an OSF and fiber Bragg gratings in a figure-8 fiber laser [28]. In 2017, we reported an ultrafast CVB laser with pulse duration of 6.87 ps using an OSF and a two-mode fiber Bragg grating (TM-FBG) as mode converter and selector, respectively [31]. In 2018, Wang et al. demonstrated an all-fiber laser generating continuous-wave CVBs by introducing a LPFG into the linear laser cavity [32]. At the same time, Zhang et al. demonstrated the switchable dual-wavelength ∼500 fs CVBs generation from a passively mode-locked fiber laser using a MSC as an output mode converter [33]. In 2019, Zeng et al. demonstrated the high-order oscillation in all few-mode fiber (FMF) laser using pump mode conversion by a MSC [34]. These works have built up the research framework of all-fiber CVB lasers. However, recent years have witnessed the bottleneck of current schemes using mode coupling theory, which seriously relies on the eigenmodes of optical fiber and, hence, lead to the biggest challenge of flexibly spatial mode controlling on demand. In the light of the powerful capabilities of metasurfaces to manipulate the light field in the spatial domain at will, therefore, metasurfaces for modulating the spatial mode in fiber lasers, in particular metafibers, is worth the wait.

In this paper, we propose a plasmonic metafiber for manipulating the spatial mode in fiber and demonstrate the direct generation of *Q*-switched CVBs from an all-fiber laser. Based on polarization-dependent plasmonic transmission of nanoholes in gold (Au) film, a polarization-independent metasurface with spatially oriented nanoholes on two-mode fiber (TMF) facet is designed to convert the fundamental mode (LP_01_) to first-order mode (LP_11_) in fiber. By using focused ion beam (FIB) lithography, the metasurface is fabricated on the TMF facet with a ceramic ferrule (CF) covered by Au film and then packaged with the single-mode fiber (SMF) with the CF using a ceramic sleeve. The mode conversion efficiency of the metafiber is measured up to 21% at the wavelengths of interest from 1529 to 1560 nm. When splicing the metafiber into an all-fiber laser cavity, the *Q*-switched CVBs, including azimuthally polarized beam (APB) and radially polarized beam (RPB), can be easily achieved at wavelength of 1548.5 nm with the assistance of a TM-FBG. The mode purities of APB and RPB are 86.5% and 90.5%, respectively. This work provides a new strategy to integrate metasurfaces into “all-in-fiber” systems and the first demonstration of the metafiber for spatial mode modulation in all-fiber lasers.

## 2 Design and fabrication of plasmonic metafiber

**Figure 1:**
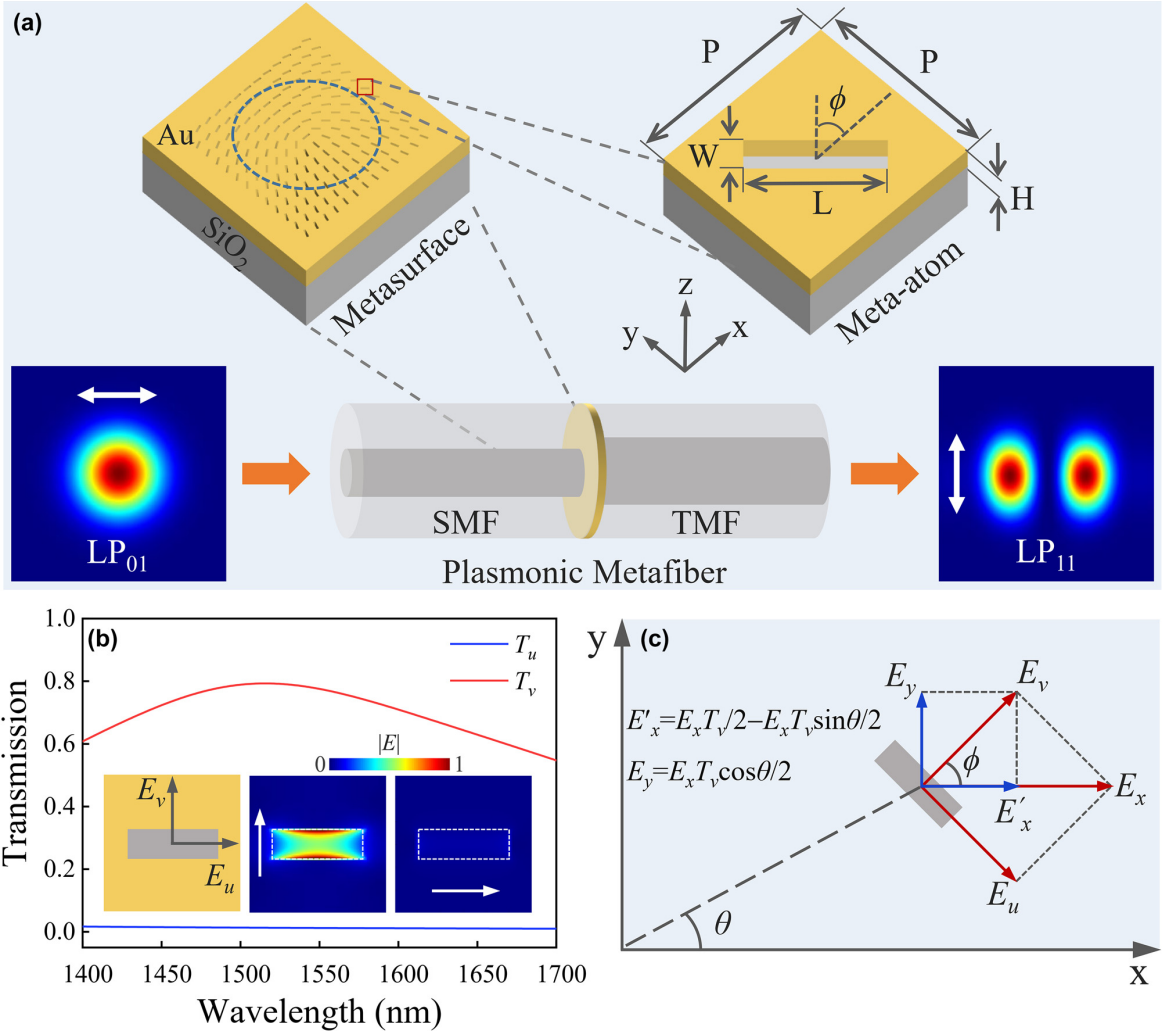
Concept, geometry, and working principle of the plasmonic metafiber. (a) Geometry of the metafiber and metasurface, and schematic of the mode conversion from LP_01_ mode to LP_11_ mode. The mode field distributions are the simulated results of metafiber. (b) Simulated transmission and electric field distributions of a single nanohole when the incident light beam is polarized along the long and short axes of nanohole. (c) Schematic of the working principle of the metasurfaces for mode conversion.

Figure 1(a) shows the concept and geometry of the plasmonic metafiber, an Au-nanohole metasurface sandwiched between a SMF and a TMF, which can convert the LP_01_ mode in SMF into LP_11_ mode in TMF. In order to excite the intrinsic LP_11_ mode in TMF, the metasurface-converted mode should exhibit similar mode profile to the LP_11_ mode. To achieve this goal, we firstly perform the finite-difference time-domain simulations of a single Au-nanohole on planar silica (SiO_2_) substrate. In the simulation, the SiO_2_ substrate is taken as a lossless dielectric with refractive index of 1.45, and the permittivity of Au film is governed by the Drude model. According to the parameter sweep results using simulations, in order to obtain the optimal efficiency at wavelength of 1550 nm, the geometric parameters of the nanoholes are selected as 420 nm in length (L), 130 nm in width (W), 50 nm in depth (H), and 600 nm in period (P). It is found that due to the polarization-dependent plasmonic resonance in Au-nanoholes, as depicted in Figure 1(b), the transmittance (*T*_*u*_) is close to 0 when exciting light is polarized along the long axis of nanohole while the transmittance (*T*_*v*_) is quite high when the polarization of exciting light is perpendicular to the long axis of nanohole at broadband infrared regime. Thus, a *x*-polarized incident light beam can be converted into a *v*-polarized light beam, working as a half-wave plate.

The operation principle of the metafiber for converting LP_01_ mode in SMF into LP_11_ mode in TMF is as follows. The spatial field distribution of the LP_11_ mode in fiber complies with 1st-order Bessel function with an additional term of the cosine function. As illustrated in Figure 1(c), due to the polarization-dependent transmittance response of the nanohole described above, the electric field of the transmission light at the location of each meta-atom is *E*_*v*_ = *E*_*x*_ *T*_*v*_cos*ϕ* when the exciting light is *x*-polarized, and its projections in the *x*-direction and *y*-direction are *E*′_*x*_ = *E*_*x*_ *T*_*v*_cos^2^ *ϕ* and *E*_*y*_ = *E*_*x*_ *T*_*v*_sin*ϕ*cos*ϕ*, respectively*.* Thus, the function of metasurface to convert the LP_01_ mode into the LP_11_-like mode can be accomplished by setting the orientation angle of each nanohole to *ϕ* = 0.5*θ* + *π*/4, as shown in Figure 1(c), where *θ* is the azimuth angle of nanoholes in polar coordinates. In such arrangement, the electric fields of output light in the *x*-direction and *y*-direction satisfy *E*′_*x*_ = *E*_*x*_ *T*_*v*_/2 − *E*_*x*_ *T*_*v*_sin*θ*/2 and *E*_*y*_ *= E*_*x*_ *T*_*v*_cos*θ*/2, respectively, where *E*_*x*_ is the electric field of *x*-polarized LP_01_ mode. It can be seen that the electric field of output light in the *y*-direction is a LP_11_-like mode and that in the *x*-direction is a superposition mode of a LP_01_ mode and a LP_11_-like mode. Under condition of *x*-polarizer, therefore, the output light of metasurface is LP_11_-like, which can successively excite the LP_11_ mode in TMF. Because of the orthogonal polarization features of generated LP_11_ and residual LP_01_ modes, it is not difficult to extract the LP_11_ mode from the two modes in TMF. To verify the performance, the model of metafiber is simulated, where the metasurface is designed using an array of 33 × 33 nanoholes with the same geometric size and different orientations in different spatial locations, obeying *ϕ* = 0.5*θ* + *π*/4. In this simulation, the substrate of metasurface is a piece of TMF and a plane wave is incident downward in SMF. The mode field distributions in Figure 1(a) clearly indicates that the LP_01_ mode in SMF is converted into the LP_11_ mode in TMF.

**Figure 2:**
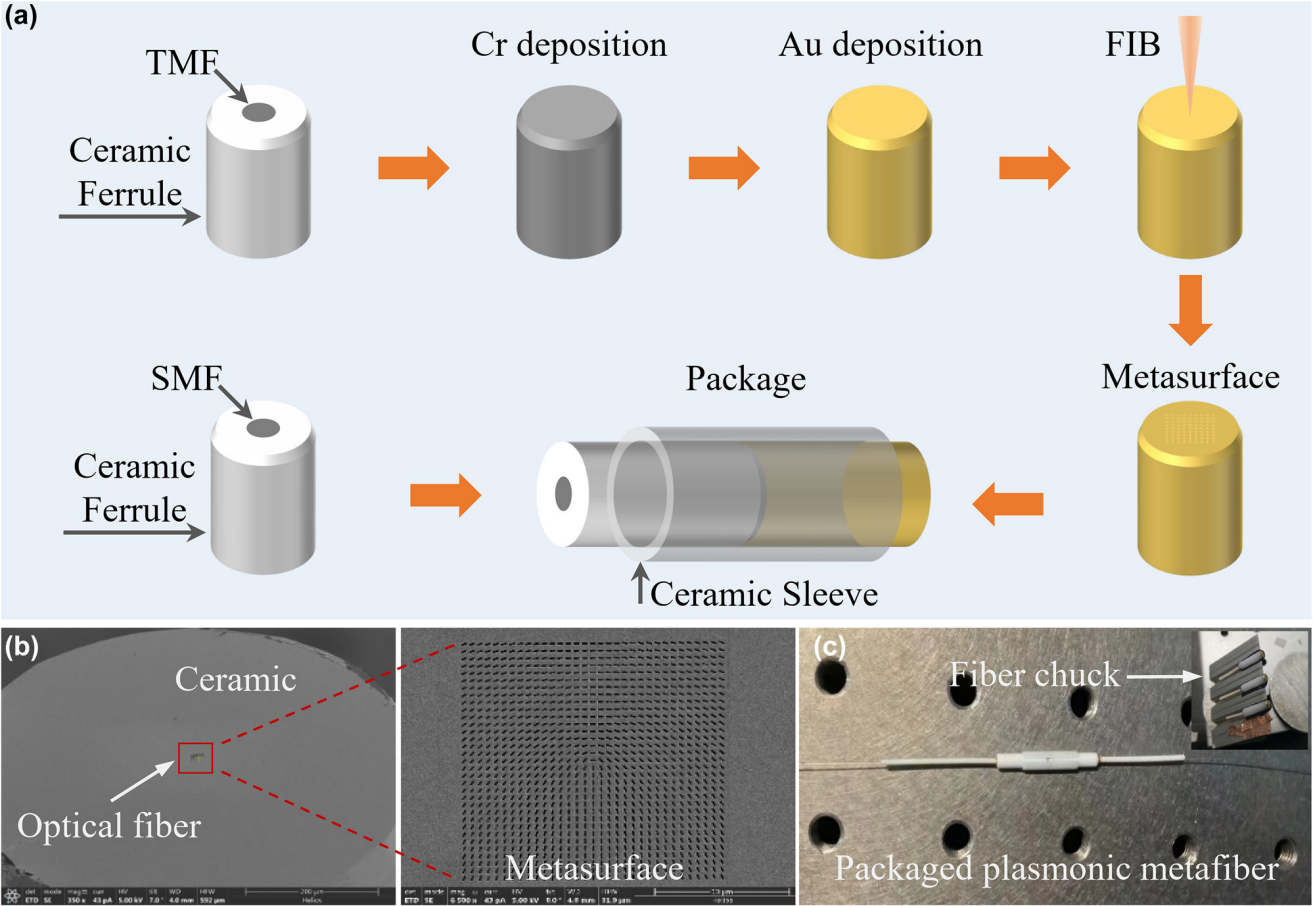
Fabrication and characterization of the plasmonic metafiber. (a) Fabrication of metasurface on TMF-CF using FIB lithography and package with SMF-CF using a ceramic sleeve. (b) SEM image of a fabricated metasurface. (c) Picture of a packaged metafiber and the fiber chuck.

The metafiber mode converter is prepared by three steps: depositing Au-film on TMF facet using thermal evaporation, fabricating an Au-nanohole metasurface on TMF facet with commercial CF using FIB lithography, and then packaging it with a SMF-CF using a ceramic sleeve. As depicted in Figure 2(a), TMF-CF and SMF-CF are firstly ready with clean and flat facet, 5-nm-thickness Cr layer and 50-nm-thickness Au layer are successively deposited on the facet of TMF-CF using thermal evaporation method. After that, the nanoholes are etched in the Au film on TMF-CF using FIB-SEM system (FEI, Helios G4 CX DualBeam). After debugging and testing, the final parameters of FIB-SEM system are set around the voltage of 30 kV, the current of 24 pA, and the working time of 2 min and 40 s. Figure 2(b) displays the scanning electron microscope (SEM) images of the fabricated metasurface on TMF-CF. The typical geometric size of nanoholes is 435 nm (L) × 135 nm (W), indicating a 4% deviation, which results from the limited accuracy of the FIB itself, the quality of the Au film, and the manual focusing of the ion beam. Due to the broadband response of the metasurface, such deviation has hardly any effect on results. Then, the TMF-CF with metasurface is packaged with a SMF-CF using a ceramic sleeve and UV-curable adhesive. The metafiber is an all-fiber component, pictured in Figure 2(c), which inherits the advantages of both fiber component and metasurface. It is note that although there exists a gap (∼10 μm) between two end-faces of CFs, in experiments, no obvious effect of the gap on laser performance is observed except for the additional loss induced by interface reflection, which can be reduced by injecting refractive-index matching liquid into the gap. In the FIB process, in order to solve the issues of platform compatibility, mechanical vibration, and electrical conductivity, a special fiber chuck is designed. As shown in the inset of Figure 2(c), this fiber chuck, made of high-conductivity and high-hardness duralumin, can ensure the quality of FIB etching on a fiber facet as high as that on a plane substrate. Compared with previously reported metafibers based on bare fibers, our metafiber based on fiber with CF can avoid the mechanical vibrations induced deviation during fabrication caused by the large aspect ratio of the bare fibers. In the package process, the CFs should be straightly inserted into the ceramic sleeve without rotation and friction when two fiber end-faces collide. This is a new strategy to integrate metasurfaces into “all-in-fiber” systems and has great potentials in novel fiber components for commercialization.

**Figure 3:**
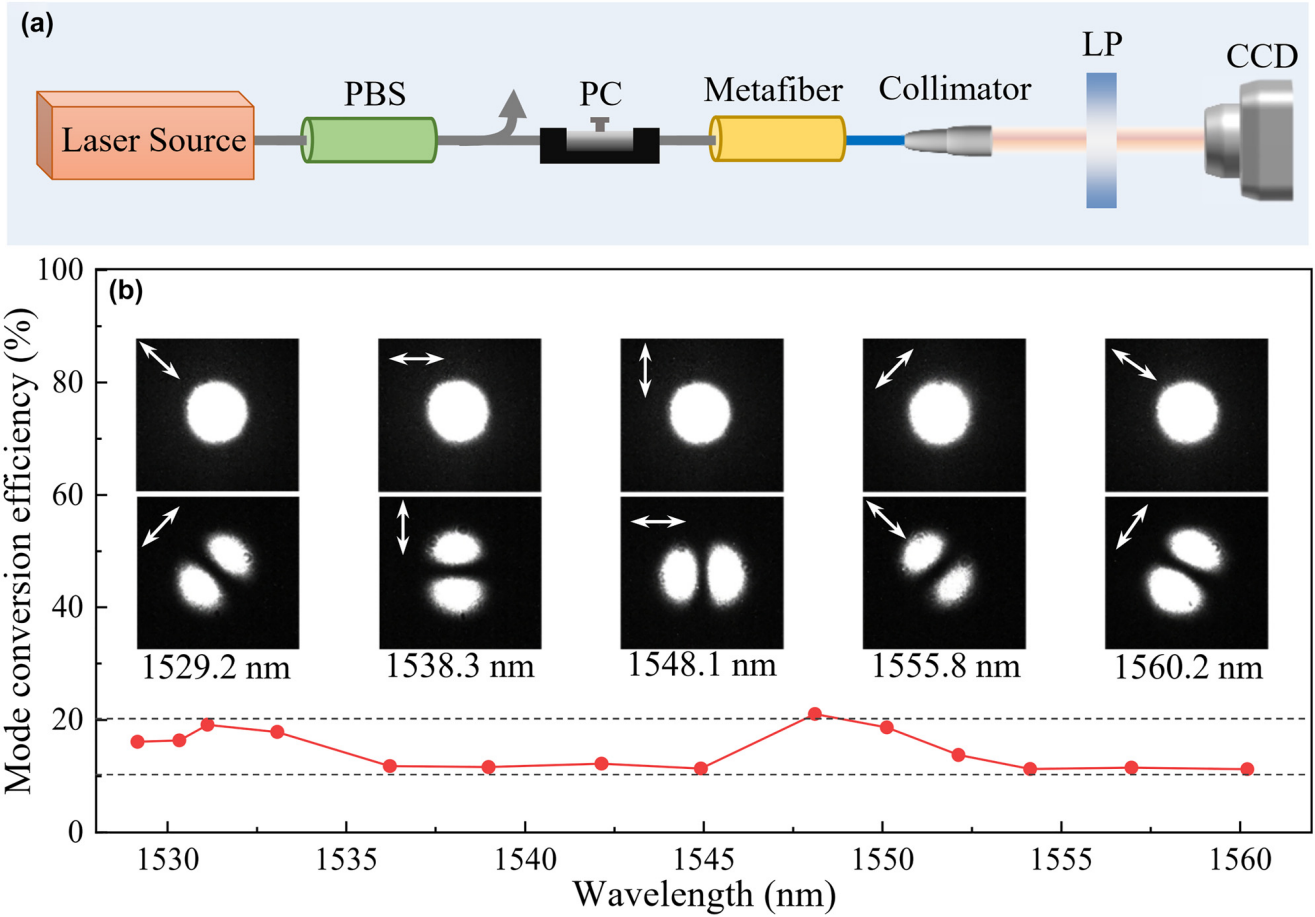
Measured mode conversion performances of the plasmonic metafiber. (a) Experimental setup for measurement. PBS, polarization beam splitter; PC, polarization controller; LP, linear polarizer; CCD, charge-coupled device. The gray and blue lines represent SMF and TMF, respectively. (b) Measured mode conversion performance and efficiency of the metafiber.

To validate the mode conversion performance of the fabricated metafiber, the output mode field and conversion efficiency are measured using an all-fiber experimental setup, as shown in Figure 3(a). In experiments, the linearly polarized LP_01_ mode in SMF is achieved by a polarization beam splitter after a wavelength-tunable laser, and a polarization controller (PC) is employed to rotate the polarization direction of the LP_01_ mode. After the mode conversion, the LP_11_ and residual LP_01_ modes output through a collimator, and then the LP_11_ mode is extracted by rotating the optical axis of the linear polarizer. The spatial intensity distributions of the LP_11_ mode are recorded by a charge-coupled device (CCD). As plotted in Figure 3(b), when the polarization direction of the LP_01_ mode is rotated by the PC, LP_11_ modes can be exited at all polarization directions, showing the feature of polarization independence. The mode conversion efficiency of the metafiber is measured from 11% to 21% at wavelengths from 1529 to 1560 nm. The conversion efficiency fluctuation in the band may result from the fabrication deviation induced geometric nonuniformity of nanoholes, which leads to different plasmonic responses at different wavelengths. The average insertion loss of the metafiber is ∼3.7 dB at wavelengths from 1529 to 1560 nm, which mainly originates from the ohmic dissipation of Au material, plasmon resonance loss of nanohole, and reflection loss of interface and Au film. Actually, the effective operation bandwidth exceeds at least 200 nm according to simulations. These results indicate that the metafiber is a broadband and polarization-independent mode converter. The experimental results agree well with the simulations and theoretical calculations.

## 3 Plasmonic metafiber-based all-fiber *Q*-switched CVB laser

**Figure 4:**
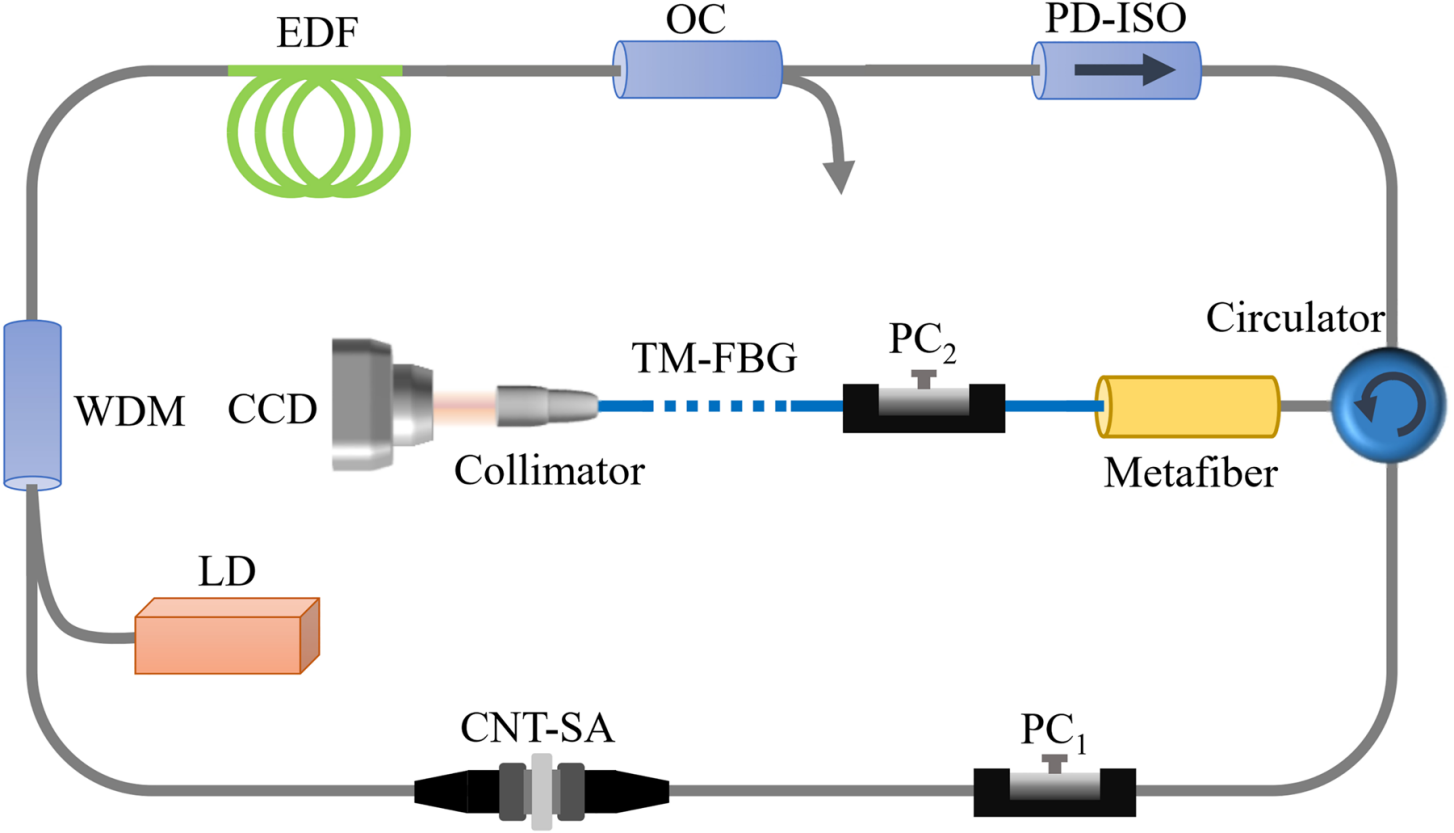
Experimental setup of all-fiber *Q*-switched CVB laser with the plasmonic metafiber. LD, laser diode; WDM, wavelength division multiplexer; EDF, erbium-doped fiber; PC, polarization controller; OC, output coupler; PD-ISO, polarization-dependent isolator; CNT-SA, carbon nanotube saturable absorber; CCD, charge-coupled device. The gray and blue lines represent SMF and TMF, respectively.

**Figure 5:**
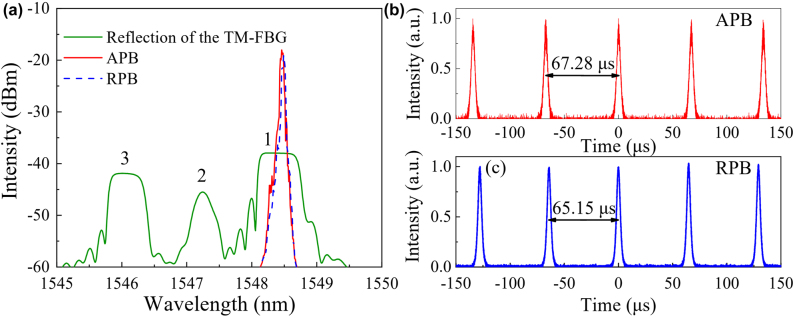
Output characteristics of the laser in spectral and temporal domains. (a) Optical spectra of the *Q*-switched APB and RPB lasers, and reflection spectrum of the TM-FBG. (b) Typical pulse train of the *Q*-switched APB laser. (c) Typical pulse train of the *Q*-switched RPB laser.

As proof of device performance, an all-fiber laser using the fabricated metafiber is conceived to generate CVBs directly from laser cavity. As illustrated in Figure 4, the laser sequentially passes through a 980/1550 wavelength division multiplexer (WDM), a 3-m erbium-doped fiber (EDF), an 80:20 output coupler (OC), a polarization-dependent isolator (PD-ISO), a 3-port circulator, a metafiber, and then is reflected by a TM-FBG and propagates through a CNT-SA. The total lengths of the SMF and TMF are 24.54 m and 0.6 m, respectively. The CVBs are exported through a collimator connected to the TM-FBG, and the spatial intensity distributions of the CVBs are recorded by the CCD. The spectral and temporal characteristics of the CVB laser are measured from the OC. In fact, after the metafiber, the LP_01_ and LP_11_ modes coexist in the TMF. As we known, the LP_11_ mode in the fiber is a scalar solution formed by the superposition of vector eigenmodes including APB and RPB. To achieve the APB and RPB from the laser, resolving and extracting them from LP_11_ mode is a next step after metafiber conversion. In our experiments, the PC_2_ and TM-FBG are adopted to achieve this goal. When the TMF is squeezed and rotated by the PC_2_, complex mode coupling happens between the vector eigenmodes caused by changing effective refractive index, polarization, and loss of the modes, and finally the CVBs including APB and RPB in TMF can be achieved. The reflection spectrum of the TM-FBG is plotted in Figure 5(a). The three reflection peaks represent the 1st to 1st order mode reflection, the 1st to 2nd order mode reflection, and the 2nd to 2nd order mode reflection, respectively. Therefore, the TM-FBG reflects the LP_01_ mode back into the ring cavity while transmits the CVBs when the laser oscillates at peak 1.

At the pump power of 20 mW, a stable continuous-wave is firstly obtained. When the pump power increases to 30 mW, the laser evolves automatically from continuous-wave state into *Q*-switched state. By adjusting the PC_2_, azimuthally or radially polarized CVBs can be observed, and the *Q*-switched state is still maintained. As depicted in Figure 5(a), the central wavelengths of the *Q*-switched APB and RPB are 1548.5 nm, which locate at the center of the reflection spectrum of the TM-FBG. It is proved that most of the LP_01_ mode power is reflected back into the fiber ring cavity, and high-quality CVBs are exported from the laser cavity. As shown in Figure 5(b) and (c), the pulse interval and pulse duration of the typical *Q*-switched APB/RPB are 67.28/65.15 μs and 4.06/3.66 μs, respectively. The fiber laser can stably emit *Q*-switched APB or RPB during the whole experiment with pump power range from 30 to 120 mW, and the spectrum and pulse trains stay almost unchanged at a fixed pump power.

**Figure 6:**
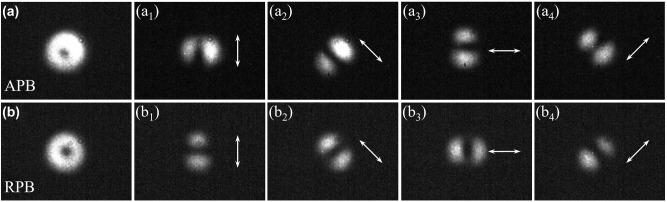
Output characteristics of the laser in spatial domain. (a) Spatial intensity distributions of the *Q-*switched APB and (b) that of the *Q-*switched RPB before and after passing a linear polarizer. The white arrows denote the transmission axis orientation of the polarizer.

In the spatial domain, the intensity distributions of the APB and RPB lasers are captured using a CCD. To further analyze the polarization state of the beam, a linear polarizer is placed in front of the CCD. Without the polarizer, as shown in Figure 6(a) and (b), the output intensities exhibit an annular intensity profile with a dark spot at the center. When passing through the polarizer at four different directions of transmission axis, the intensity distributions are shown in Figure 6(a_1_–a_4_) and (b_1_–b_4_). The intensity distribution direction of the passed light rotates with the transmission axis of the polarizer, denoted by white arrows in Figure 6, and the dark bands are always parallel/perpendicular to the transmission axis of the polarizer, indicating that the output laser beam is APB/RPB. Such *Q*-switched APB and RPB states can be easily switched by adjusting the PC_2_.

**Figure 7:**
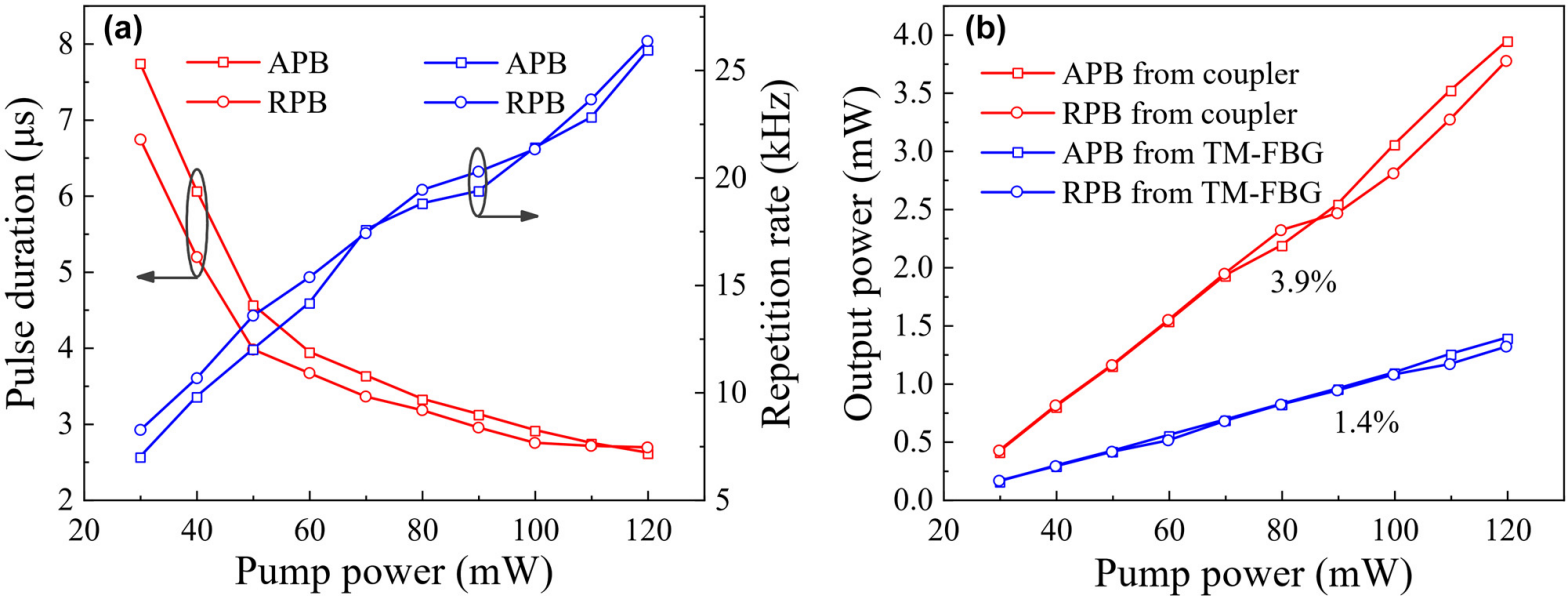
Evolution of the *Q-*switched CVB laser with pump power. (a) Pulse duration and repetition rate. (b) Output power from OC and collimator.

When the pump power varies from 30 to 120 mW, the laser maintains stable *Q*-switched operation. Figure 7(a) gives the pulse duration and repetition rate evolutions as a function of pump power. It can be observed that the pulse duration of the *Q*-switched APB/RPB decreases with increasing the pump power while the repetition rate increases, which are the typical features of *Q*-switched lasers. The output power of *Q*-switched APB/RPB from OC and collimator is recorded for comparison, as shown in Figure 7(b). The threshold and slope efficiency of the *Q*-switched CVB laser are 30 mW and 1.4%, respectively. Considering that the output ratio of the OC is 20%, the highest efficiencies of mode conversion from the LP_01_ mode to APB/RPB are calculated to be 9.37%/9.55%, calculated by the ratio of the output power of CVBs to the total power in the cavity. It should be noted that after replacing the metafiber with an unpatterned TMF-CF, the laser can only output the LP_01_ mode from both ports when the laser still oscillates at peak 1, indicating that the metafiber plays a well-defined mode conversion role in the laser. The mode purities of the *Q*-switched APB and RPB lasers are measured by the tight bend approach, which is based on the difference of bending loss between the fundamental mode and the second-order modes in fiber [35]. When the laser works at RPB mode, the output power is measured as 721 μW without bending and 67 μW when the fiber is bent to a pencil under the same pump power. Thus, the mode purity of the RPB is estimated to be 90.7%. Similarly, the mode purity of the APB is 86.5%. It should be pointed that although the mode conversion efficiency of metafiber is below 21%, the high reflectivity of TM-FBG (∼98.6%) for the fundamental mode can guarantee high purity of first-order modes at output port. The imperfect spatial intensity distributions in Figure 6 are mainly attributed to the defect of CCD.

Compared with the traditional fiber mode converters, including OSF, LPFG, and MSC, the metafiber shows several advantages and potentials. The OSF technique, based on lateral offset mode exciting from SMF to FMF, strongly depend on the lateral offset distance (sub-micrometer order), which is simple but hard to accomplish for advanced fiber fusion splicer. In principle, the coupling efficiency is theoretically limited to 20.7% [31], thus leading to large loss and strong polarization dependence. In addition, the OSF is fragile and easily broken at the offset splicing spot, which limits its practical applications. The LPFG technique provides mode conversion between fundamental mode and high-order modes in FMF, such as laser inscribed [32], acoustically induced [36], and pressure induced [37]. In contrast to the acoustically induced and pressure-induced LPFGs, which render the laser system more complicate, laser-inscribed LPFGs is simple. However, the rather narrow optical bandwidth of LPFG make it difficult to generate pulsed and even ultrafast CVB lasers, which usually requires large bandwidth [30]. The MSC is based on the phase (propagation constants) matching between the fundamental mode in SMF and high-order modes in FMF, which is realized by fused tapered fiber coupling. When the phase matching condition is satisfied well, the MSC exhibits high conversion efficiency and broad conversion bandwidth in theory [33]. However, in reality, the high-precision controlling of tapered fiber diameter and fusing is difficult for fabricating MSC by using fused tapered fiber system. In addition, it is difficult to couple fundamental mode to even high-order modes due to large phase mismatching and also difficult to directly excite vector modes in fiber. In comparison, the metafiber has advantages of high-precision controllability, good reproducibility, broad optical bandwidth, and polarization independence, which provides an elegant solution to the difficulties of traditional fiber mode converters. Although the experimentally measured efficiency is currently below 21%, it is believed that the efficiency can be further improved by optimizing design and fabrication, such as Au-nanostrip metasurface and all-dielectric metasurface [38, 39]. It is also experimentally measured that even at input of the 20 mW, 500 fs laser, no obvious damage is observed for our metafiber, showing good stability against optical damage. The results demonstrate that the proposed metafiber can be an alternative solution to modulate the spatial mode in all-fiber lasers.

## 4 Conclusions

We have proposed a plasmonic metafiber for converting LP_01_ mode to LP_11_ mode in fiber and demonstrated an all-fiber *Q*-switched CVB laser using the metafiber. Based on the polarization-dependent transmission of nanohole in Au film induced by plasmonic resonance, a polarization-independent mode conversion metasurface with spatially oriented nanoholes is designed and fabricated on TMF-CF facet covered by Au film using FIB lithography. Then, the metafiber is prepared as an all-fiber component by packaging with the SMF-CF using a ceramic sleeve, and the mode conversion efficiency of the metafiber is up to 21% at measured wavelengths from 1529 to 1560 nm. To reduce the insertion loss, and hence improve the efficiency, adopting the complementary structure, *i.e.*, nanostrip, is the first path. The other potential solutions are adding refractive-index matching liquid during packaging to reduce the interface reflection loss; using electron beam evaporation or magnetron sputtering to improve the quality of Au film; enlarging the footprint of Au-metasurface; and replacing SMF with polarization-maintaining optical fiber as the input. To greatly reduce the loss and improve the efficiency, all-dielectric metasurface is the best route [38, 39]. However, the direct fabrication of all-dielectric metasurface on fiber facet is still challenging currently. In the subsequent studies, we will probe into the aforementioned issues.

In contrast to metasurfaces on plane substrates, our metafiber shows excellent interface compatibility with fiber optic systems, thus providing the feasibility of introducing the metasurfaces into the all-fiber lasers to modulate the spatial modes. As proof of application, the metafiber is spliced into an all-fiber laser cavity, and finally the *Q*-switched CVBs, including APB and RPB, have been easily achieved. The mode purities of APB and RPB are 86.5% and 90.7%, respectively. Note that, the mode-locking state is not observed yet in experiments. It can be attributed to the large cavity loss induced by the metafiber and the narrow reflection bandwidth of the TM-FBG. Compared with previously reported fiber mode converters, the proposed metafiber exhibits lots of advantages including broadband, polarization-independent, high efficiency, high optical damage, and high-precisely controllability using nano-manufacturing technology, showing great potential in commercialization of a novel all-fiber component. Furthermore, this work provides a new strategy to integrate metasurfaces into “all-in-fiber” systems and offers a reliable method to modulate the spatial modes in all-fiber lasers. Although it is demonstrated in TMF, the proposed method and strategy can be extended to FMF system, which supports other high-order modes. We believe that, in the future, the metafiber family will produce its advantages in light field manipulations in spatial, temporal, and spatiotemporal domains to infuse new life and capabilities into all-fiber lasers [40–42].
